# Understanding and addressing female pelvic pain ‐ a multifaceted challenge

**DOI:** 10.1111/aogs.14682

**Published:** 2023-09-29

**Authors:** Tina Tellum, Davor Jurkovic

**Affiliations:** ^1^ Department of Gynecology Oslo University Hospital Oslo Norway; ^2^ Gynecology Diagnostic and Outpatient Treatment Unit University College London Hospital London UK

Chronic pelvic pain (CPP) is a major health issue which blights the lives of one in five women around the world.^1^ This persistent and often debilitating condition can have a profound negative impact on the quality of life for women which affects their physical, emotional, and social well‐being. The broader societal implications include the economic burden through lost workdays and healthcare costs, not to mention the strain on relationships and families which many women experience as a result of CPP.^2^

Pelvic pain can represent a diagnostic challenge as multiple pelvic pain syndromes often overlap in the same patient, as well as nonpelvic conditions, such as migraine, chronic fatigue, and fibromyalgia.^3^ Not only gynecologists, but a range of different healthcare professionals meet people with pelvic pain. Asking specialists about which condition comes to mind first when thinking of pelvic pain, you will likely receive a myriad of responses – from endometriosis to pelvic floor disorders to biosocial models, each expert offering a perspective colored by their background and special interest.

While this diversity of viewpoints is expected, it might represent a problem when field‐specific blinders are put on. This might inadvertently lead to misdiagnoses and, worse yet, disregard for patients' experiences. The consequences of such oversight are significant, as undiagnosed conditions can result in patients being dismissed,^4^ or inappropriate and possible harmful treatments initiated. On the other hand, medical practitioners may often find it hard to discern the root causes of CPP and decide on the most appropriate treatment.

In this themed issue, we have brought together studies with a broad range of interests and expertise to cover various aspects of pelvic pain, aiming to bring awareness and broaden perspectives to this complex subject.

Endometriosis stands as a leading cause of chronic pelvic pain. Three studies in this issue look at deep‐ and bowel endometriosis from different perspectives. While Chaggar et al.^5^ demonstrate the reproducibility of ultrasound predictors important for optimizing surgery planning, Hudelist et al.^6^ show the association of different surgical techniques for bowel endometriosis with pelvic pain and bowel function. Knez et al.^7^ on the other hand complete the perspective by investigating what happens when deep endometriosis is left untreated ‐ will it automatically progress, as often feared by doctors and patients? The answer is no, demonstrating that expectant management can the best “treatment” for the right patient. What can be done for the patient suffering from endometriosis associated pain, who has no benefit from either surgery or medication? A broad grasp of pain mechanisms, including central sensitization, mandates that physicians adopt a more holistic perspective. Sandström et al.^8^ report that altered GABAa receptor function might play a role in endometriosis associated pain, inviting us to look beyond the pelvis to find possible new treatment strategies.

Women who suffer from pelvic pain often look towards pregnancy as a temporary shelter from pain. However, this phase can be overshadowed by pelvic girdle pain. Several new works published in this issue provide new insight into risk factors for developing pregnancy‐related pelvic pain. In the study by Ertmann et al.,^9^ involving 1491 women, it was discovered that the most influential predictor of pregnancy‐related pain in the second and third trimesters was the experience of similar pain in the first trimester. Depressive symptoms identified early in pregnancy were linked to the onset and severity of pelvic girdle pain later in the pregnancy in the prospective study conducted by Algård et al.^10^ Lastly, a study involving 356 women revealed that while generalized joint hypermobility did not heighten the risk of pelvic girdle pain during or post‐pregnancy, those with such hypermobility combined with a higher body mass index did report greater pain intensity early in their pregnancy (Ahlqvist et al.).^11^ Health care providers should be encouraged to remain vigilant to these risk factors in early pregnancy and proactively initiate measures to prevent the exacerbation of these pelvic pains.

Transitioning from the joys of childbirth, we must also recognize its potential to cause pelvic pain through obstetrical injuries. Huber et al.^12^ used three‐dimensional endoanal ultrasonography (3D‐EAUS) to study postpartum anal sphincter defects and found not only that these were often overlooked, but that they showed a significant correlation with perineal pain and dyspareunia. This not only suggests the utility of 3D‐EAUS in postnatal follow‐ups but underlines once more the significance of prevention of obstetric injuries.^13^ In another study in this themed issue, perineal reconstruction post‐childbirth significantly alleviated pelvic floor symptoms.^14^

The qualitative study by Myrtveit‐Stensrud et al.^15^ is unique as it looks at both individuals with vulvodynia and their partners. Vulvodynia, with its characteristic “burning” or “knife‐like” pain, remains enigmatic, and little has been done to understand the effect on couples until now. This oversight in research makes their work especially noteworthy. The study reveals that heterosexual couples face challenges in grasping the nature of vulvodynia, struggling with understanding the pain that impacts their social and sexual lives. The introduced fear‐avoidance‐endurance model by Myrtveit‐Stensrud et al. sheds light on feelings of powerlessness, loneliness, guilt, and shame. Improved communication is essential to address these challenges.

Simultaneously, the role of patient narratives, psychological evaluations, and pain mapping cannot be underestimated. Saga et al.^16^ translated and modified a comprehensive tool that could be useful for many medical professionals to monitor and assess patients suffering from pelvic pain.

In conclusion, as we delve into the intricate layers of female pelvic pain, it becomes obvious that a siloed approach is obsolete. The complex interplay of physiological, psychological, and social factors demands a multidisciplinary care approach, encompassing gynecologists, physiotherapists, psychologists, and pain management specialists. By fostering this collaborative approach to care, we should be able to help better many women grappling with the adversities caused by chronic pelvic pain.

## CONFLICT OF INTEREST STATEMENT

TT reports receiving personal fees for lectures on ultrasound from GE Healthcare, Samsung, Medtronic and Merck, outside of the scope of this work. DJ reports no conflict of interest.

## References

[1] Ahangari A. Prevalence of chronic pelvic pain among women: an updated review. Pain Physician 2014;17:E141‐7. 24658485

[2] Mathias SD , Kuppermann M , Liberman RF , Lipschutz RC , Steege JF . Chronic pelvic pain: prevalence, health‐related quality of life, and economic correlates. Obstet Gynecol 1996;87:321–7. 8598948 10.1016/0029-7844(95)00458-0

[3] Lamvu G , Carrillo J , Ouyang C , Rapkin A . Chronic pelvic pain in women: a review. Jama 2021;325:2381–91. 34128995 10.1001/jama.2021.2631

[4] Hoffman SR , Farland LV , Doll KM , Nicholson WK , Wright MA , Robinson WR . The epidemiology of gynaecologic health: contemporary opportunities and challenges. J Epidemiol Community Health 2020:75, 398, 401. 10.1136/jech-2019-213149 PMC8095335 33109525

[5] Chaggar P , Tellum T , Cohen A , Jurkovic D . Intra‐ and interobserver reproducibility of transvaginal ultrasound for the detection and measurement of endometriotic lesions of the bowel. Acta Obstet Gynecol Scand. 2023;102:1306‐1315. 10.1111/aogs.14660 PMC10541154 37641421

[6] Hudelist G , Pashkunova D , Darici E , et al. Pain, gastrointestinal function and fertility outcomes of modified nerve‐vessel sparing segmental and full thickness discoid resection for deep colorectal endometriosis—A prospective cohort study. Acta Obstet Gynecol Scand. 2023;102:1347‐1358. 10.1111/aogs.14676 PMC10541157 37694901

[7] Knez J , Bean E , Nijjar S , Tellum T , Chaggar P , Jurkovic D . Natural progression of deep pelvic endometriosis in women who opt for expectant management. Acta Obstet Gynecol Scand. 2023;102:1298‐1305. 10.1111/aogs.14491 PMC10540915 37190782

[8] Sandström A , Bixo M , Bäckström T , Möller A , Turkmen S . Altered GABAA receptor function in women with endometriosis: a possible pain‐related mechanism. Acta Obstet Gynecol Scand. 2023;102:1316‐1322. 10.1111/aogs.14559 PMC10541155 36944570

[9] Ertmann RK , Nicolaisdottir DR , Siersma V , et al. Factors in early pregnancy predicting pregnancy‐related pain in the second and third trimester. Acta Obstet Gynecol Scand. 2023;102:1269‐1280. 10.1111/aogs.14670 PMC10541159 37771202

[10] Algård T , Kalliokoski P , Ahlqvist K , Schlager A , Kristiansson P . Role of depressive symptoms on the development of pelvic girdle pain in pregnancy: A prospective inception cohort study. Acta Obstet Gynecol Scand. 2023;102:1281‐1289. 10.1111/aogs.14562 PMC10541153 36965059

[11] Ahlqvist K , Bjelland EK , Pingel R , et al. Generalized joint hypermobility and the risk of pregnancy‐related pelvic girdle pain: Is body mass index of importance?—A prospective cohort study. Acta Obstet Gynecol Scand. 2023;102:1259‐1268. 10.1111/aogs.14664 PMC10540924 37614096

[12] Huber M , Larsson C , Lehmann JP , Strigård K , Lindam A , Tunón K . Sonographic postpartum anal sphincter defects and the association with pelvic floor pain and dyspareunia. Acta Obstet Gynecol Scand. 2023;102:1290‐1297. 10.1111/aogs.14606 PMC10540925 37350333

[13] Aasheim V , Nilsen ABV , Reinar LM , Lukasse M . Perineal techniques during the second stage of labour for reducing perineal trauma. Cochrane Database Syst Rev 2017:Cd006672, 6. 10.1002/14651858.CD006672.pub3 PMC6481402 28608597

[14] Rotstein E , Ullemar V , Engberg H , Lindén Hirschberg A , Ajne G , Tegerstedt G . One‐year follow‐up after standardized perineal reconstruction in women with deficient perineum after vaginal delivery. Acta Obstet Gynecol Scand. 2023;102:1338‐1346. 10.1111/aogs.14666 PMC10540923 37594200

[15] Myrtveit‐Stensrud L , Haugstad GK , Rème SE , Schaller SL , Groven KS . “It's all my fault”: a qualitative study of how heterosexual couples experience living with vulvodynia. Acta Obstet Gynecol Scand. 2023;102:1378‐1389. 10.1111/aogs.14537 PMC10540927 36879489

[16] Saga S , Stafne SN , Dons V , Bratlie I , Spetalen S , Hagemann CT . Amsterdam complex pelvic pain symptom scale with subscales: Based on a Norwegian translation, psychometric assessment and modification of the Amsterdam hyperactive pelvic floor scale. Acta Obstet Gynecol Scand. 2023;102:1409‐1423. 10.1111/aogs.14665 PMC10540920 37675780

